# Fluorescent aminal linked porous organic polymer for reversible iodine capture and sensing

**DOI:** 10.1038/s41598-020-72697-x

**Published:** 2020-09-29

**Authors:** Muhammad A. Sabri, Mohammad H. Al-Sayah, Susan Sen, Taleb H. Ibrahim, Oussama M. El-Kadri

**Affiliations:** 1 Department of Chemical Engineering, American University of Sharjah, P.O. Box 26666, Sharjah, United Arab Emirates; 2 Department of Biology, Chemistry, and Environmental Sciences, American University of Sharjah, P.O. Box 26666, Sharjah, United Arab Emirates

**Keywords:** Materials science, Polymer chemistry, Environmental impact

## Abstract

A novel triazene-anthracene-based fluorescent aminal linked porous organic polymer (TALPOP) was prepared via metal free-Schiff base polycondensation reaction of 9,10-bis-(4,6-diamino-*S*-triazin-2-yl)anthracene and 2-furaldehyde. The polymer has exceptional chemical and thermal stabilities and exhibit good porosity with Brunauer–Emmett–Teller surface area of 401 m^2^g^−1^. The combination of such porosity along with the highly conjugated heteroatom-rich framework enabled the polymer to exhibit exceptional iodine vapor uptake of up to 314 wt % and reversible iodine adsorption in solution. Because of the inclusion of the anthracene moieties, the TALPOP exhibited excellent detection sensitivity towards iodine via florescence quenching with *K*_sv_ value of 2.9 × 10^3^ L mol^−1^. The cost effective TALPOP along with its high uptake and sensing of iodine, make it an ideal material for environmental remediation.

## Introduction

Nuclear energy is becoming one of the most feasible alternative sources to meet the ever-increasing energy demand and minimize the emission of greenhouse gases because of its high-density energy, minimal carbon footprints, and low operation cost^1–4^. Despite such advantages, the potential emissions of radioactive material (such as ^129^I and ^131^I, ^3^H, ^14^CO_2_, and ^85^Kr) from nuclear energy power plants is a major drawback of this technology due to the serious environmental and health effect of these materials^4,5^. The long-lived radionuclides of iodine, ^129^I (half-life of 1.57 × 10^7^ years) and ^131^I (half-life of ca. 8 days), are usually emitted in the gas form which then enters the food chain through contaminated air or by depositing into soil and water. The radioactive iodine isotopes have adverse effects on human metabolic system and health, and they were indicated as major cause of thyroid cancer^5–7^. Accordingly, there is an urgent need to develop new technology and means to effectively detect, capture, and store radioactive iodine.

In this context, several adsorbent materials have been studied and tested for effective sequestration and sensing of iodine^4,5,8–13^. It has been reported that iodine sequestration efficiency is a function of several structural properties of the adsorbent including surface area, pore size, specific high-affinity binding sites, polar groups, and conjugated units. Thus, increasing the affinity of the host to iodine, in addition to enhanced surface area, can have tremendous effect on iodine capture^4,5,14,15^. Materials like activated carbon, silica, silver-doped zeolites, chalcogenide aerogels, and microporous polymers have been reported to show good adsorption capacities for radioiodine^5,16,17^. However, most of these have several shortcomings such as limited surface area, high cost, low sensitivity to iodine, and difficulty in regeneration that make them quite unsuitable for practical usage^5,18–21^. For example, silver-doped zeolites are expensive and have been reported to have low adsorption capacities for iodine while metal organic frameworks, in general, are unstable in humid conditions^18–22^.

Porous organic polymers (POPs), on the other hand, are another class of solid porous materials that have found applications in variety of areas that include gas storage and separation, catalysis, supercapacitors, light harvesting, and iodine capture^4,7,19,23–25^. POPs are getting increasing attention for iodine capture due to their high surface areas, versatility in design, tunable pore size and pore volumes, excellent thermal and chemical stability, and high physiochemical robustness. As such, the chemical structures of POPs can be tailor-made to incorporate heteroatoms with expanded conjugated systems in which the lone pairs on heteroatoms and the conjugated surfaces account for strong interactions between the polymers and iodine^12,15,26,27^. For example, Liu et al. reported the preparation of novel thiophene-based porous organic networks that can capture up to 204 wt.% of iodine^28^. More recently, Pan et al. developed N- and S-rich covalent organic framework (COFs) possessing high iodine uptake of 276 wt.%^29^. Triazine-based conjugated polymers have also been reported to have respectable iodine uptake, presumably due to their nitrogen-rich nature^4,15,30–35^. The high number of basic nitrogen sites on the triazine unit enhances the uptake capacity that originates from the formation of charge-transfer complexes of nitrogen-iodine (donor–acceptor) systems formed during iodine adsorption on the polymers^7,12,15,36^. Geng et al. constructed a series of novel triazine-based conjugated microprous polymers (TCMPs) applying the Friedel–Crafts polymerization reactions^12,32,37,38^. The TCMPs showed remarkable iodine uptake and exhibited high detection sensitivity toward nitroaromatic compounds.

Nevertheless, despite the many reported adsorbent materials for iodine capture and storage, needs still exist for the development of new materials that are economically viable and can be prepared in a large scale with a minimal laborious synthetic protocols. Accordingly and building up on our recent reports on the use of aminal linked porous organic polymers (ALPOPs) in iodine capture and metal sensing^4,39^, we envision to design and prepare multifunctional, low-cost, heteroatom-rich, and luminescent aminal linked porous organic polymer by combining nitrogen-rich triazene building block that compromises an anthracene moiety and a heterocyclic aldehyde linker. Herein, we report on the synthesis of a triazine-anthracene aminal linked porous organic polymer (TALPOP) by reacting 9,10-bis-(4,6-diamino-S-triazin-2-yl)anthracene (DTA), the core of the envisioned POP, and 2-furaldehyde, as the organic linker. The DTA building block not only offers the nitrogen-rich nature, but it also includes anthracene unit, which provides additional π-conjugated structures in the TALPOP polymeric framework, leading to positive impact on the iodine adsorption and provides good fluorescence and emission properties , which makes it a potential candidate for the development of aqueous fluorescent chemical sensors for iodine. The aldehyde linker (2-furaldehyde) is commercially available, inexpensive, and introduces additional heteroatom, thus making the resulting TALPOP ideally and economically feasible material for iodine capture and storage. The iodine uptake, release, regeneration, and adsorption kinetics were studied and reported in this work. The chemically and thermally stable novel polymer showed excellent iodine vapor uptake up to 314 wt.% and was able to release over 90% of the loaded iodine when immersed in ethanol. The TALPOP can be recycled over five times, at least, without significant loss of iodine uptake capacity. In addition, it exhibited remarkable detection sensitivity towards iodine through fluorescence quenching in dioxane with *K*_sv_ values as high as 2.9 × 10^3^ L mol^−1^.

## Experimental section

### Materials, instrumentation, and methods

DTA was prepared according to previously published procedure^40^. All other starting materials and solvents were purchased from Sigma-Aldrich and used directly as received without further purification. A EuroEA3000 series elemental analyzer was used to determine the percentage content of H, C, and N in the polymer. ^1^H NMR spectrum of the synthesized starting material (DTA) was obtained using a Bruker-400 MHz NMR spectrometer. Fourier transfer infrared (FTIR) spectroscopy was performed on a Perkin-Elmer spectrometer having an attenuated total reflectance accessory. Thermogravimetric analysis (TGA) was performed using a Perkin-Elmer thermogravimetric analyzer under oxygen atmosphere with a heating rate of 5 °C min^−1^ and a temperature range of 30–800 °C. To obtain Scanning Electron Microscope (SEM) images of the polymer, samples were prepared by dispersing the polymer onto a sticky carbon tape attached to an aluminum sample holder. The SEM images were taken by TESCAN-LMU SEM. Powder X-ray diffraction (PXRD) patterns were collected in 2θ range of 5–50 at room temperature and scan speed of 5° min^-1^ using a Panalytical X’pert pro multipurpose diffractometer with a Cu Kα radiation. The textural properties were obtained by carrying out nitrogen adsorption–desorption experiments using a Quantachrome Autosorb iQ volumetric analyzer at 77 K. Samples weighing approximately 40 mg were degassed for 12 h at 120 °C and vacuum prior to the sorption measurements. The Brunauer–Emmett–Teller (BET) method and the non-local density functional theory (NLDFT) were used to calculate the surface area and pore size distribution. Shimadzu UV-1800 spectrometer was used to carry out UV–Vis measurements. Iodine loading capacity was determined by exposing a specific amount (40–50 mg) of the polymer in a glass vial to iodine vapor at 350 K and ambient pressure and measuring its weight before and after exposer. Weight percentages of iodine uptake were calculated by applying the following equation: $\frac{{\mathrm{m}}_{2}-{\mathrm{m}}_{1}}{{\mathrm{m}}_{1}} \times 100\mathrm{ wt}.\mathrm{\%},$ where m_1_ and m_2_ are masses of polymer powder before and after iodine loading, respectively. The adsorption kinetics of iodine on TALPOP (1 mg/mL) was studied at 25 °C by monitoring the removal of iodine from cyclohexane solutions at different concentrations (100 and 300 mg L^−1^). On the other hand, the binding isotherms of TALPOP were generated by monitoring the amount of iodine adsorbed as the concentration of iodine in cyclohexane increased. Samples of the polymer (1 mg/mL) were soaked in iodine solutions with different concentrations (100, 200, 300, 400 and 500 ppm) for 72 h. The concentrations of iodine remaining in the solutions were then determined using UV–Vis absorption at 523 nm and based on calibration curves. Finally, the ability of polymer to release its encapsulated iodine was studied by placing a given amount (5 mg) of the iodine-loaded-polymer (I_2_@TALPOP) in ethanol (15 mL) and the amount of the released iodine was monitored using UV–Vis spectroscopy. Fluorescence spectra were obtained using Edinburgh Instrument, FLS-900 scanning spectrofluorometer. The polymer was dispersed in dioxane (1 mg/10 mL) and the resulting suspension was sonicated for about 30 min at room temperature. Fluorescence spectra (λ_ex_ = 389 nm) were recorded after aliquots of iodine solution in dioxane (10 mM) were added to a quartz cuvette containing 2 mL of the suspension.

### Synthesis of TALPOP

To a 50-mL round bottom flask equipped with a condenser and magnetic stir bar, DTA (400 mg, 1.01 mmol), 2-furaldehyde (400 mg, 4.16 mmol), and DMSO (12 mL) were added. The mixture was degassed by bubbling argon for 15 min and then stirred for 3 days under argon gas at 180 °C. After cooling to room temperature, the dark precipitate was isolated using medium glass frit and subsequently washed with tetrahydrofuran (THF), acetone, methanol, and dichloromethane, and then dried under vacuum at 120 °C to afford a dark brown colored powder, TALPOP, with 64% yield. Anal. Calcd. for C_40_H_32_N_10_O_4_: C, 67.03%; H, 4.50%; N, 19.54% ; Found C, 67.41%; H, 5.60%; N, 21.00%.

## Results and discussion

### Synthesis and characterization

**Scheme 1:**
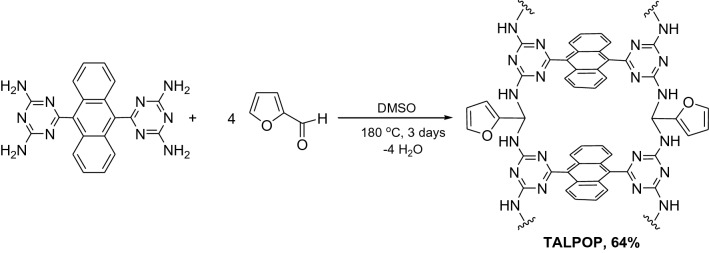
Synthetic route for the preparation of the TALPOP.

TALPOP was prepared by one-pot-Schiff base condensation reaction in high yields as dark brown powder by treating DTA with four equivalents of 2-furaldehyde in DMSO under argon atmosphere at refluxing temperature for three days (Scheme 1). The straightforward synthetic route is metal-free and does not require the addition of a catalyst. This is of significant importance not only from environmental point of view but also for economical and practical reasons. Moreover, the utilization of the commercially available, inexpensive, and biomass-based 2-furaldehyde, adds another advantage to the partially “biomass-derived” TALPOP.

The prepared TALPOP is insoluble in water, diluted acid (HCl), diluted base (NaOH), or common organic solvents such as acetone, chloroform (CHCl_3_), acetonitrile, THF, dimethylformamide (DMF), 1,4-dioxane (DOX), and ethanol. Such insolubility hints to high degree of cross-linked structure and high chemical and physical stability in different environments and allows for their purification.

**Figure 1:**
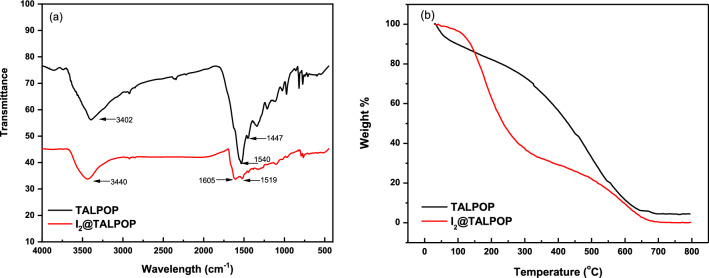
FT-IR spectrum **(a)** and TGA plots **(b)** of TALPOP and I_2_@TALPOP.

The structure, chemical connectivity, functional groups, crystallinity, morphology, thermal stability, and composition of TALPOP were determined using spectroscopic and analytical techniques such as FTIR, PXRD, SEM, TGA, and elemental analysis. FTIR spectra (Fig. 1a) suggests the successful formation of the TALPOP by showing a band at 3402 cm^−1^ assigned to the stretching frequency of the secondary amine (NH) and 1540 and 1447 cm^−1^ (quadrant and semicircle stretching) corresponding to the triazine ring. Moreover, the absence of the carbonyl groups (C=O) of the aldehydes at 1685 cm^−1^ and primary amine (NH_2_) at 3469 cm^−1^ indicate the consumption of the functional groups (Figure S1). It is well-documented that the Schiff base reaction between amino and aldehyde functional group leads to imine (C=N) bonds. However, if the basicity of the amino groups is enhanced by electron rich substituent, for example triazene as the case in DTA, further reaction can take place between the formed imine bond and the amino groups to from stable aminal bonds (HN–CHR–NH)^7,41^. The fact that the FTIR spectra does not show peaks at around 1600 cm^−1^ (C=N stretching vibration) and the presence of N–H stretching peak at 3402 cm^−1^, suggests the formation of aminal linkage between the DTA and 2-furaldehyde. Such linkage formation has been confirmed for many POPs involving amino substituted triazene and aldehydes by the use of FTIR and solid-state ^13^C NMR techniques^7,41,42^. The synthesized TALPOP is amorphous and lack any long-range order as confirmed by PXRD (Figure S2). The amorphous nature is a well-known property for many POPs^23^. SEM images of the polymer revealed aggregates of irregular-block like structures (Figure S3 (b)). The thermal stability was assessed by TGA analysis of pristine TALPOP implying excellent thermal stability of the polymer up to ~ 350 °C (Fig. 1b). Initially, a weight loss of ~ 15% was observed at temperatures up to 100 °C attributed to the release of adsorbed water. This step was followed by a slow-rate of weight loss (~ 10%) up to 350 °C, attributed to the release of trapped solvent molecules in the pores of the polymer, before the onset of polymer degradation.

### Porosity study

**Figure 2:**
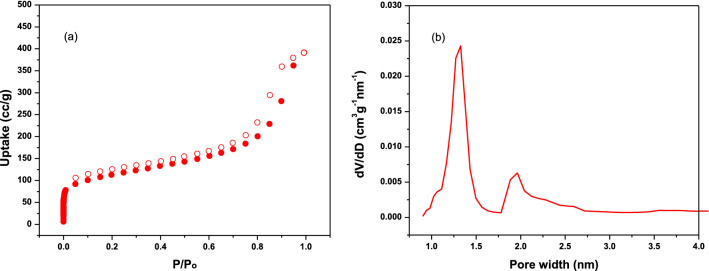
Nitrogen adsorption (filled spheres)-desorption (hollow spheres) isotherms **(a)** and pore size distribution **(b)** of TALPOP at 77 K.

Pore characteristics and specific surface area of the TALPOP were determined through nitrogen adsorption–desorption isotherm at 77 K (Fig. 2a). The isotherm showed a steep increase in the nitrogen gas uptake at low relative pressure (P/Po < 0.01), and slower uptake at the 0.01–0.8 P/Po range, indicating the material is mostly microporous with minor mesoporous pore size distribution (PSD)^43^. The BET surface area of 401 m^2^g^−1^ is comparable to many reported aminal linked porous organic polymers^7,33,39^. PSD (1.3 nm and 1.96 nm) and pore volume (0.871 cm^3^g^−1^ at P/Po = 0.99) were determined using the non-local density functional theory (NLDFT) (Fig. 2b). The less than 2 nm PSD confirms the existence of a highly microporous structure.

### Iodine vapor capture

**Figure 3:**
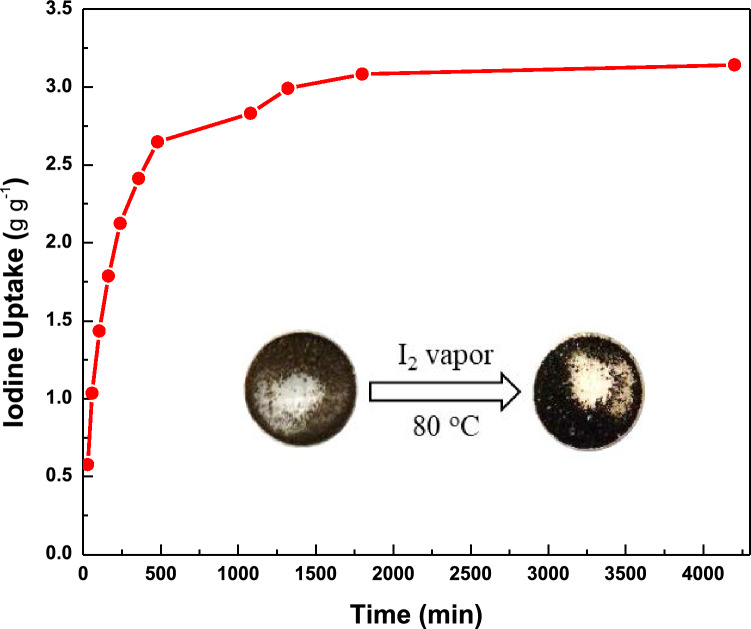
Gravimetric iodine uptake by TALPOP over time at 350 K and ambient pressure. Inset: photographs showing color change after the exposure TALPOP to iodine vapor.

In order to study the iodine capture using TALPOP, pre-weighed dried samples and excess amount of solid iodine were placed in a glass vial at 80 °C and ambient pressure in a closed system. These conditions (80 °C and ambient pressure) depict a typical nuclear fuel reprocessing unit conditions^13,37^. The amount of iodine adsorbed on the TALPOP was determined by weighing the samples at different time intervals. Figure 3 shows the iodine capture by TALPOP over a period of about three days; the results show that iodine capture was rapid in the first 8 h, after which it slowed down. There was negligible change in iodine loading weights after 24 h, suggesting saturation of the TALPOP. It is quite interesting to note that as the iodine capture by TALPOP proceeded, the color of the sample changed from brown to black (Fig. 3 inset) indicating iodine encapsulation into the polymer’s framework. The equilibrium uptake of iodine by TALPOP was determined to be 3.14 g g^−1^. The remarkable iodine uptake is among the highest value reported to date for ALPOPs^4,34,39,44^. We attribute this high uptake to the rich-heteroatom framework, large pore volume, and the large conjugated porous network represented by the anthracene moieties. To gain more insight of the interactions between the iodine molecules and the TALPOP framework, FTIR, TGA, and PXRD studies were carried out for I_2_@TALPOP. The FTIR spectra showed that the band at 3402 cm^−1^ assigned to secondary amine (NH) shifted to 3440 cm^−1^ while the stretching frequencies of the triazine ring at 1540 and 1447 cm^−1^ changed to 1605 and 1519 cm^−1^ (Fig. 1), respectively. Such changes clearly indicate a strong interaction between the iodine molecules and TALPOP framework. Characteristic peaks of elemental iodine were not observed in the PXRD spectra (Figure S2) and thus suggesting that the iodine molecules are uniformly distributed in the framework of the TALPOP. Such uniformity was also confirmed by SEM images of the I_2_@TALPOP (Figure S3 (b)). Moreover, no noticeable changes in the pristine TALPOP morphology after performing iodine uptake experiment suggesting that the iodine molecules are confined in the pores of the TALPOP. The TGA of I_2_@TALPOP indicated a major weight loss between 90 and 350 °C (Fig. 1b). Since the pristine TALPOP is stable at this temperature range, it can be concluded that the weight loss is due to the release of iodine from the I_2_@TALPOP (Fig. 1b). The 69 wt.% estimated iodine loss at 350 °C relative to the I_2_@TALPOP is consistent with the TALPOP iodine uptake capacity where at 3.14 g g^−1^ loading capacity, 75 wt.% of I_2_@TALPOP is iodine. It is also worth noting that the insignificant change in weight of I_2_@TALPOP at temperatures below 100 °C (as compared to pristine TALPOP) indicates that there is insignificant amount of water adsorbed, suggesting more preference for iodine adsorption over water by the polymer.

### Iodine adsorption from solution

**Figure 4:**
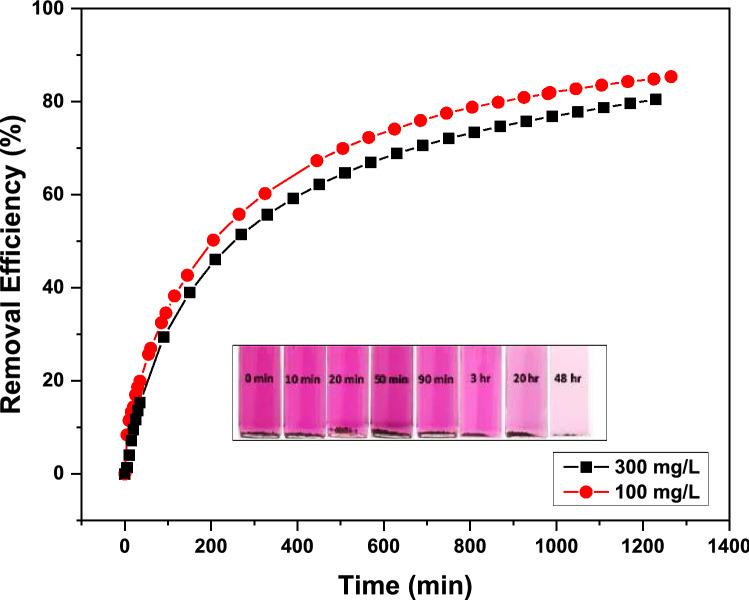
Kinetic studies of iodine adsorption by TALPOP in cyclohexane solution. Insert: photograph showing the change in color at different time intervals.

In addition to iodine vapor capture, the TALPOP can also adsorb iodine from iodine-cyclohexane solution. Pre-weighted amounts (3 mg) of TALPOP were added to iodine solutions (3.0 mL at 100 mg L^−1^ or 300 mg L^−1^) in small sealed vails, and the purple colored solution faded away to very light pink over time (Fig. 4 insert) indicating the removal of iodine from the solutions. The residual amounts of iodine were monitored using UV–Vis spectroscopy. Figure 4 reveals two-stage adsorption kinetics of iodine by the TALPOP in cyclohexane solution. In stage 1, adsorption was quite rapid in the initial few hours (2–4 h) presumably due to the availability and exposure of many active sites on the polymer surface. In stage 2, there was a slow increase in the adsorption of iodine as the pores and active sites became further occupied (especially on the polymer surfaces) by iodine until equilibrium was reached. Removal efficiencies of 86% (86 mg/g uptake) and 81% (243 mg/g uptake) were recorded after 20 h for the 100 and 300 mg L^−1^ iodine-cyclohexane solutions, respectively. Such results suggest that the TALPOP is not only efficient, but also fast in removing iodine from solutions. It is obvious that the higher the initial iodine concentration, the faster the iodine uptake by the polymer, presumably due to the greater collision probability between the iodine molecules and the polymer’s active sites^45^. However, the lower removal efficiency at higher initial iodine concentration is possibly due to the saturation of the pores and active sites by the large amount of iodine molecules in solution.

**Figure 5:**
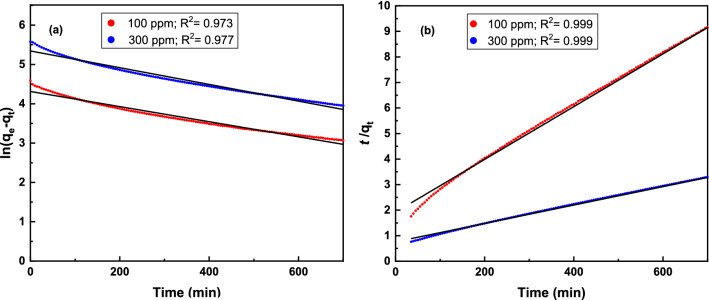
Pseudo First order kinetics **(a)** and pseudo second order kinetics, **(b)** of iodine adsorption on TALPOP from iodine-cyclohexane solution. The points represent the experimental data at the corresponding concentrations (100 mg/L and 300 mg/L) and the solid lines represent the corresponding model fit (temperature: 298 K; adsorbent dosage: 1 mg/mL).

To further understand the kinetics of the adsorption process, the change in iodine concentration in solution over time was fitted to pseudo-first order and pseudo-second order kinetic models at 100 mg L^−1^ and 300 mg L^−1^ initial iodine concentrations (Fig. 5). The correlation coefficient *R*^2^ values for the pseudo-first order and pseudo-second order kinetic were determined to be 0.973 and 0.999 (Table 1, S4), respectively. This suggests that the adsorption kinetics of TALPOP follows the pseudo-second order kinetic model, which indicates a chemisorption-type process^39^. Such conclusion is consistent with the presence of high interaction between the polymer surface and the iodine molecules. The existence of electron-rich heteroatoms (N, O) and aromatic rings in the polymer skeleton provide electron-donating moieties that are capable of donating electrons to the electron accepting iodine, leading to charge-transfer (CT) interaction and enhancement in iodine enrichment^46^.

**Table 1:** Kinetic parameters of pseudo-first order and pseudo-second order kinetic models.

		Initial concentration (mg g^−1^)	
		100	300
**Kinetic model**	*q*_e_ (Exp) (mg g^−1^)	97.9	264.2
**Pseudo-1st order**	*q*_e_ (mg g^−1^)	74.6 ± 0.8	209 ± 2
	*k*_1_ (g mg^−1^ min^−1^)	1.92 × 10^–3^ ± 2.7 × 10^–5^	2.13 × 10^–3^ ± 2.7 × 10^–5^
	*R*^2^	0.973	0.977
**Pseudo-2nd order**	q_e_ (mg g^−1^)	96.9 ± 0.2	277.8 ± 0.6
	*k*_2_ (g mg^−1^ min^−1^)	5.53 × 10^–5^ ± 4.6 × 10^–7^	1.70 × 10^–5^ ± 1.2 × 10^–7^
	*R*^2^	0.999	0.999

*q*_e_ adsorbed amount at equilibrium, *k*_1_ and *k*_2_ are the pseudo-first-order and pseudo-second-order constants, respectively, of the adsorption process.

**Figure 6:**
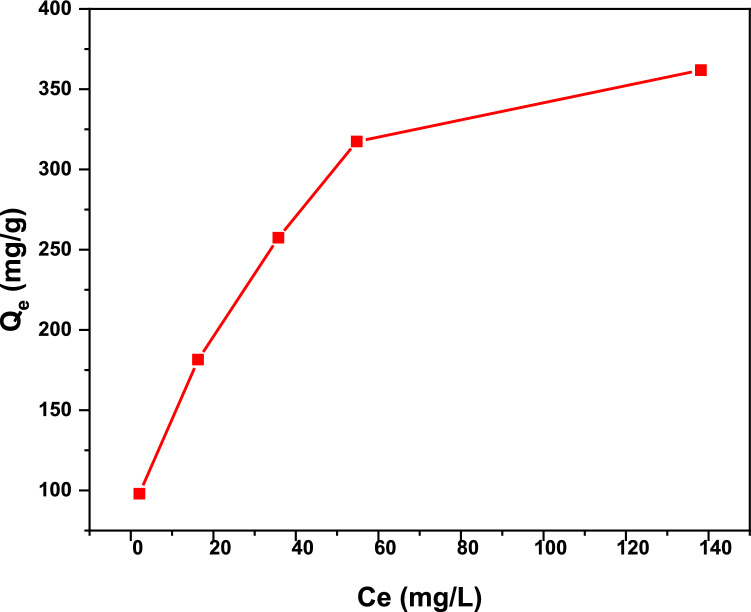
The adsorption isotherm of TALPOP over iodine initial concentration of 100–500 mg/L at 298 K and adsorbent dosage of 1 mg/mL.

**Figure 7:**
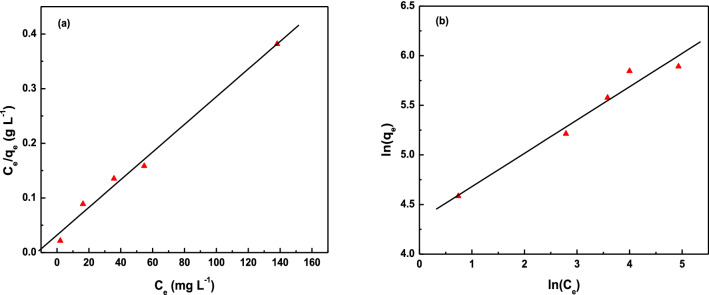
Fitting of adsorption isotherm data of TALPOP to Langmuir **(a)** and Freundlich **(b)** models.

Adsorption isotherm for the removal of iodine by TALPOP was also constructed over an iodine initial concentration of 100–500 mg L^−1^ and at a temperature of 25 °C (Fig. 6). After soaking for 72 h with TALPOP (1 mg/mL), the residual iodine concentrations in the solutions was measured using UV–Vis spectroscopy. The obtained isotherm data (Fig. 7) was fitted to both Langmuir and Freundlich models; the former is used to model adsorption through homogenous monolayer process, while the latter is used to examine the heterogenous multilayer adsorption (S 4)^47,48^. The Langmuir isotherm (Fig. 7a) was obtained for the polymer by plotting *Ce*/*Qe* vs *Ce*, where *Ce* is the equilibrium concentration of iodine (mg/L) and *Qe* is the amount of iodine adsorbed per gram of TALPOP at equilibrium (mg/g). On the other hand, the Freundlich model (Fig. 7b) was applied by plotting ln(*Qe*) vs ln(*Ce*) to explore the possibility for multilayer adsorption process with reversibility.

The adsorption parameters are summarized in Table 2. The linear correlation coefficient value (*R*^2^) of the Langmuir model is higher as compared to that of Freundlich model suggesting a monolayer adsorption process, presumably due to the large aromatic π-surface area. The initial part of the isotherm in Fig. 6 increases almost vertically, indicating a strong TALPOP-iodine affinity at lower concentrations to reach a maximum adsorption capacity (Q_m_) calculated to be 400 mg of iodine per gram of polymer^45^.

**Table 2:** Adsorption parameters of isotherm models for iodine adsorption on TALPOP from solution.

Isotherm	Parameters	
**Langmuir**	K_L_ (L/mg)	7.79 × 10^–2^
	*Q*_m_ (mg/g)	400
	*R*^2^	0.989
**Freundlich**	K_f_ (mg^(1–1/n)^L^1/n^/g)	76.9
	N	2.97
	*R*^2^	0.964

Langmiur model: Q_m_ = maximum monolayer coverage capacity (mg/g) K_L_ = Langmuir isotherm constant (L/mg) Freundlich model: K_f_ = Freundlich isotherm constant (mg/g).

### Iodine release and TALPOP regeneration

**Figure 8:**
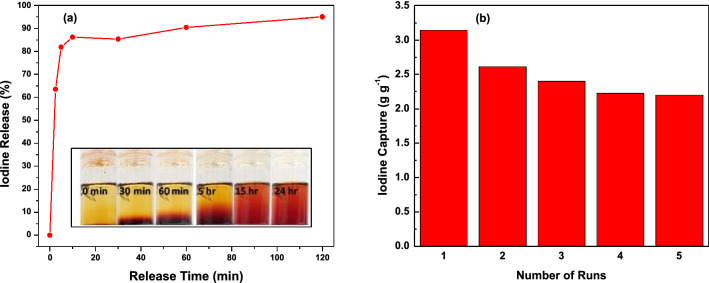
Release of iodine from TALPOP by immersion in ethanol **(a)** and the change in TALPOP efficiency for iodine capture by vapor sublimation upon recycling **(b)**.

In order to test the recyclability of TALPOP, iodine release and polymer regeneration were studied using ethanol as the organic solvent. A known quantity of I_2_@TALPOP was immersed in a known volume of ethanol for two hours at room temperature. The color of the solution changed quickly from colorless to dark brown indicating iodine transfer from the iodine encapsulated in TALPOP to ethanol (Fig. 8a, insert). The release of iodine into ethanol was monitored using UV–Vis spectra over different time intervals. It was observed that ~ 85% of the adsorbed iodine was released within the first 20 min and almost all of the encapsulated iodine was released after two hours of incubation in ethanol (Fig. 8a). These results indicate that captured iodine can be easily removed from I_2_@TALPOP by immersing it in ethanol and the polymer can be regenerated for another cycle of iodine adsorption. Thus, to determine the recyclability of TALPOP, a regenerated sample which, showed insignificant discoloration (Figure S4) as compared to the pristine TALPOP, was reused over five cycles for gaseous iodine uptake using similar conditions (as mentioned above). After the first cycle of adsorption-regeneration, the adsorption efficiency of the recovered TALPOP decreased by ~ 15% and only 2–3% for each of the subsequent four cycles (Fig. 8b). Thus, it can be concluded that the TALPOP can retain up to 80% of its initial iodine capture capacity after five cycles, which is comparable with many other reported POPs^26,34,39^. This also demonstrates that the TALPOP can be efficiently recycled and repeatedly utilized over many cycles without significant decline in iodine capturing capacity. Such results make TALPOP an excellent adsorbent material for practical application in the removal of radioactive iodine and provides guidelines for the design and preparation of novel POPs as adsorbents for environmental remediation.

### Fluorescence sensing for iodine

**Figure 9:**
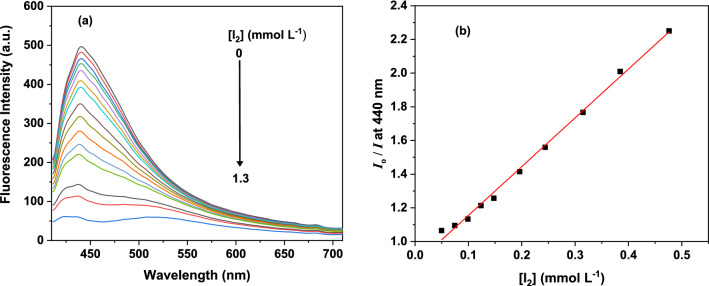
Fluorescent spectra (λex = 389 nm) of TALPOP upon titrating with iodine solution (0.01 mol L^−1^) **(a)** and Stern–Volmer model fit of the relative change of fluorescent at 440 nm as iodine concentration increases **(b)**.

The presence of the anthracene unit within the framework of TALPOP makes the polymer fluorescent and provides an optical probe for detecting the interaction of the guest (iodine) with the polymer. The interaction of iodine with the conjugated surface and the heteroatoms alters the electronics of the system and, hence influence the emission profile of the polymer. Thus, in order to determine the potential applications of TALPOP as fluorescent sensor for iodine in solution, samples of the polymer (0.1 mg/mL) were dispersed by sonication in some common organic solvents such as ethanol, chloroform, THF, acetone, acetonitrile, DMF, and DOX^37,38,49^. The fluorescence spectra (Figure S5) of the TALPOP is highly dependent on the solvent molecules, presumably due to the interaction of the solvent molecules with the π-conjugation structure of the TALPOP, leading to a reduced aggregation^38,50^. Among these solvents, TALPOP showed the greatest fluorescent intensity when it was dispersed in DOX (Figure S5)^37,38^ and accordingly it was used as the medium for the polymer fluorescence studies. We monitored the effect of iodine concentration in DOX on the emission of the polymer. Millimolar concentrations of iodine solutions were titrated into a uniform suspension of TALPOP in DOX (0.1 mg/mL) and the fluorescence spectra of the polymers were scanned after each addition. A systematic instantaneous decrease in the emission of TALPOP (Fig. 9a) was observed upon increase in iodine concentration. The fluorescence spectra showed a significant decrease (> 90%) in the emission as the concentration of iodine in solution increased to above 0.01 M (Fig. 9a). Thus, in order to quantify the quenching effect of iodine on the emission of the polymer, we applied the Stern–Volmer equation to determine the quenching coefficient. The Stern–Volmer plot show a good correlation between the concentration of iodine (the quencher) and the relative change in fluorescence intensity (at 440 nm) of TALPOP (Fig. 9b). The quenching coefficient (*K*_sv_) for iodine was estimated to be 2.9 × 10^3^ L mol^−1^ (S5), which is comparable with those reported for many POPs for fluorescence sensing of iodine^12,32,38^. An electron-transfer mechanism could explain the fluorescence quenching of the TALPOP. The electron-rich building block of the polymer (electron donor) and the low-lying-LUMOs of iodine (− 4.99 eV, electron acceptor), provide a driving force for electron transfer and hence quench the fluorescence^51^.

## Conclusions

A novel fluorescent aminal linked porous polymer was prepared via Schiff-base condensation reaction. The resulting polymer exhibits remarkable thermal and physiochemical stability presumably due to the rich π-conjugated structure. Because of such properties along with the large surface area and electron-rich nature, the polymer can capture a significant amount of iodine (3.14 g g^−1^). The encapsulated iodine molecules can be easily released once the loaded polymer is immersed in polar solvent. The desorbed polymer showed excellent reloading capacity over 5 cycles. The aminal linked polymer also capable of removing over 85% of iodine from cyclohexane solution. In addition, it possesses excellent sensing sensitivity toward iodine with *K*_sv_ value of 2.9 × 10^3^ L mol^−1^. Results from this work suggest that aminal linked polymers are promising materials for multi-environmental applications.

## Supplementary information

Supplementary Information.
