# Supplementary material for: Fluorescent aminal linked porous organic polymer for reversible iodine capture and sensing
Source: Sci Rep. 2020 Sep 29;10:15943. doi: 10.1038/s41598-020-72697-x (PMC7525493; doi:10.1038/s41598-020-72697-x)
Supplement: Supplementary file 1 — Supplementary Information. [file 41598_2020_72697_MOESM1_ESM.pdf]

Electronic Supporting Information

Fluorescent aminal linked porous organic polymer for reversible iodine capture and sensing

Muhammad A. Sabri,[1] Mohammad H. Al-Sayah,[2] Susan Sen,[2] Taleb H. Ibrahim,[1] Oussama M. El-Kadri*[2]

[1]Department of Chemical Engineering

[2]Department of Biology, Chemistry, and Environmental Sciences

American University of Sharjah,

P.O. Box 26666, Sharjah, United Arab Emirates

*Corresponding author, Tel:+97165152787

Email address: oelkadri@aus.edu

Table of Contents

I. Characterization

- FTIR
- Powder X-ray
- SEM

II. Iodine Adsorption Kinetics

- Rate equations

III. Adsorption Isotherms

IV. Fluorescent Quenching of TALPOP

- Fluorescent spectra of TALPOP in different solvents
- Stern-Volmer Binding Equation

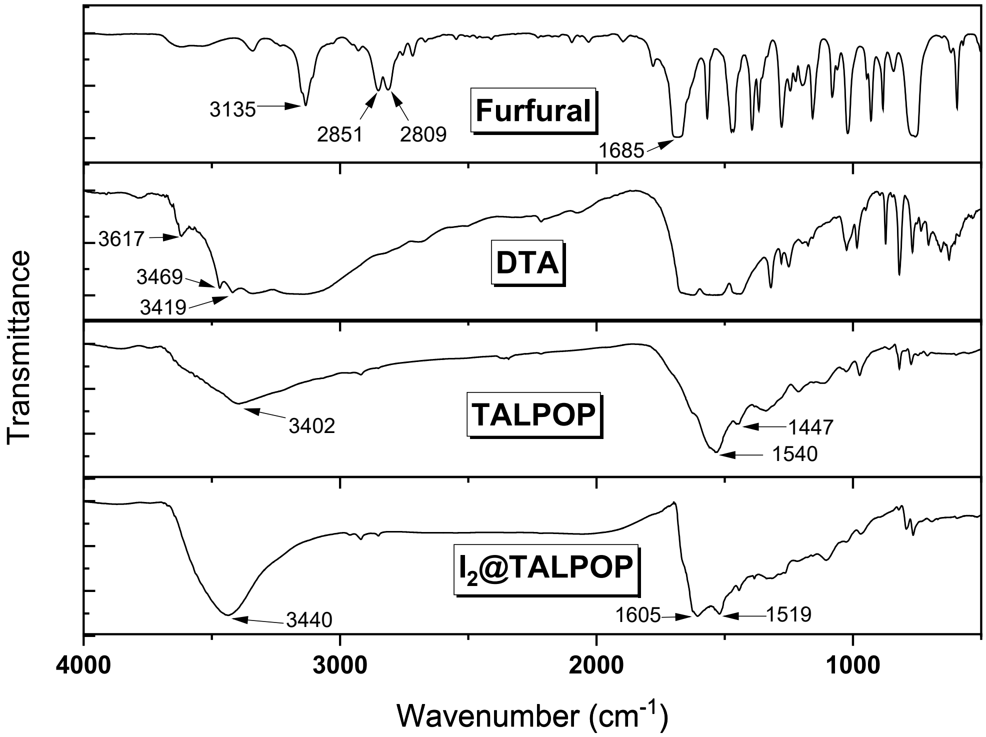

Figure S1: FTIR of the starting materials, **TALPOP**, and **I$_2$@TALPOP**

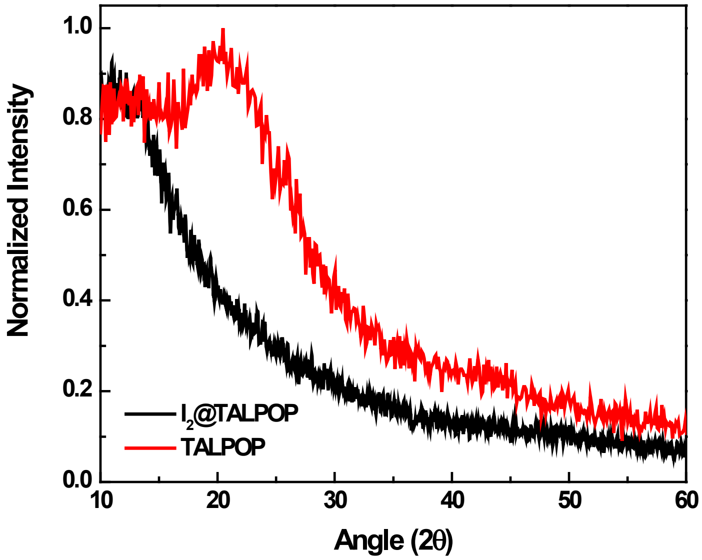

Figure S2: Powder X-ray diffraction patterns of **TALPOP** and **I$_2$@TALPOP**

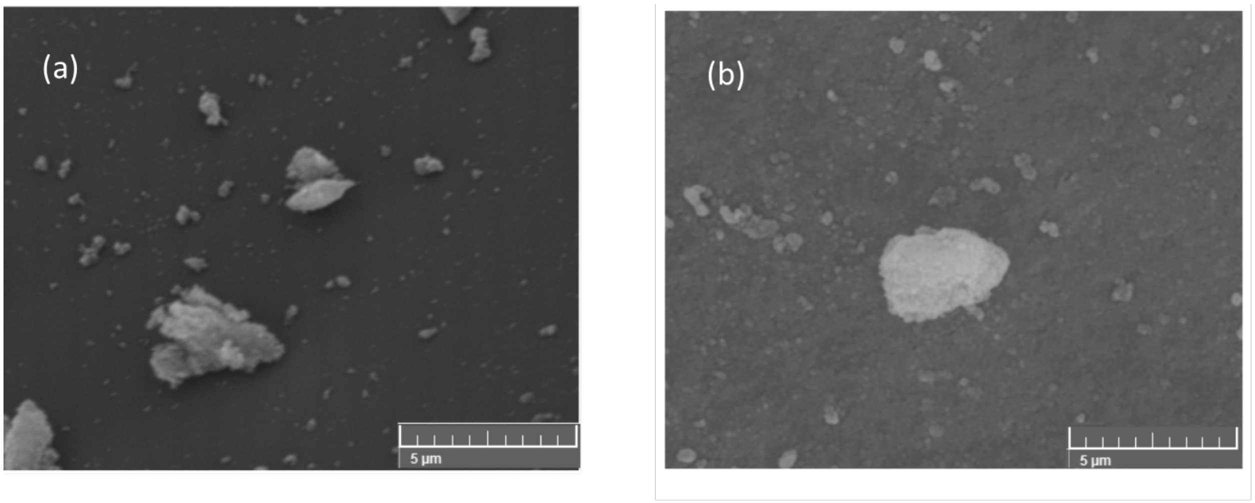

Figure S3: SEM images of **TALPOP** (a) and **I_2@TALPOP** (b)

Iodine Adsorption Kinetics

The adsorption kinetics of iodine by the TALPO were studied at 25 °C by monitoring the removal of iodine from cyclohexane solutions at different concentrations (100 ppm and 300 ppm). The obtained data fitted with very good correlation to pseudo-second order kinetic model according to equation II (below).

The equations of these kinetic models are:[1]

$$\ln[q_e - q(t)] = \ln q_e - k_1 t$$ (I) for pseudo-first order and

$$\frac{t}{q(t)} = \frac{t}{q_e} + \frac{1}{k_2 q_e^2}$$ (II) for pseudo-second order equation

where q_t is the amount of adsorbed iodine, q_e is the amount of adsorbed iodine at equilibrium, k_1 is the pseudo-first order rate constant, k_2 is the pseudo-second order rate constant, and t is the contact time. Table 1 is the kinetic parameters of pseudo-first order and pseudo-second order kinetic models.

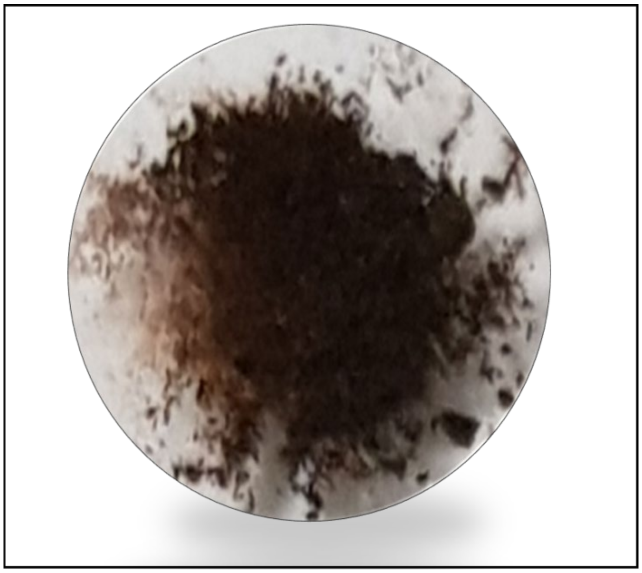

Figure S4: Photo of **TALPOP** after regeneration.

Adsorption isotherm

Langmuir and Freundlich isotherm

Langmuir model was linearly fitted by plotting C_e/Q_e vs Ce according to the following equation:

$$\frac{C_e}{Q_e} = \frac{1}{Q_m K_L} + \frac{C_e}{Q_m}$$

where: C_e = the equilibrium concentration of adsorbate (mg/L), Q_e = the amount of iodine per gram of the adsorbent at equilibrium (mg/g). Q_m = maximum monolayer coverage capacity (mg/g) K_L = Langmuir isotherm constant (L/mg).

Freundlich isotherm model were linearly fitted by plotting (ln Q_e) Vs (ln C_e), according to Freundlich equation:

$$\ln Q_e = \ln K_f + \frac{1}{n} \ln C_e$$

where K_f = Freundlich isotherm constant (mg/g) n = adsorption intensity; C_e = the equilibrium concentration of adsorbate (mg/L), Q_e = the amount of iodine adsorbed per gram of the adsorbent at equilibrium (mg/g).

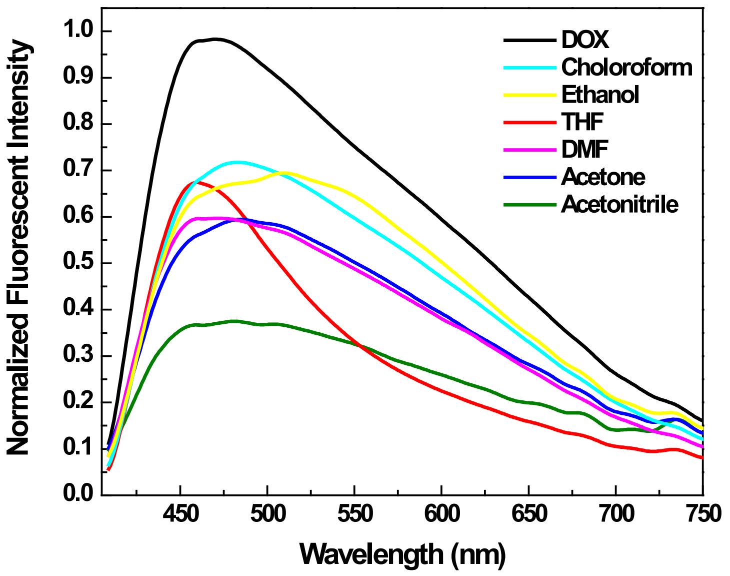

Figure S5: Fluorescent spectra (λex = 389 nm) of **TALPOP** in different solvents.

Stern-Volmer plot: The stern-Volmer plot for the relative change in the emission of the polymers versus the increase in the concentration of iodine shows a linear trend for the quenching of the fluorescence by the iodine (Figure 9B)

$$\frac{Io}{I} = 1 + Ksv\,[M]$$

Where I_o is the fluorescence intensity of the polymer without iodine addition. I is the fluorescence intensity after adding iodine [M], and Ksv is the quenching coefficient.[2,3]

References

1. Simonin, J. P. On the comparison of pseudo-first order and pseudo-second order rate laws in the modeling of adsorption kinetics. *Chem. Eng. J.* **300**, 254–263 (2016).

2. Thomas, S. W., Joly, G. D. & Swager, T. M. Chemical sensors based on amplifying fluorescent conjugated polymers. *Chem. Rev.* **107**, 1339–1386 (2007).

3. Abdelmoaty, Y. H., Tessema, T. D., Choudhury, F. A., El-Kadri, O. M. & El-Kaderi, H. M. Nitrogen-Rich Porous Polymers for Carbon Dioxide and Iodine Sequestration for Environmental Remediation. *ACS Appl. Mater. Interfaces* **10**, 16049–16058 (2018).
